# Diagnosis of persistent ovarian carcinoma with three-step immunoscintigraphy

**DOI:** 10.1054/bjoc.1999.0972

**Published:** 2000-01-18

**Authors:** P Magnani, F Fazio, C Grana, C Songini, L Frigerio, S Pecorelli, G Mangili, N Colombo, C D A Mariani, G Paganelli

**Affiliations:** INB-CNR, Institute H. S. Raffaele, University of Milan, Milan, Italy; University of Brescia, Milan, Italy; European Institute of Oncology, via Ripamonti 435, I-20132 Milan, Italy

**Keywords:** ovarian carcinoma, monoclonal antibodies, avidin–biotin

## Abstract

The diagnosis of recurrent ovarian carcinoma is usually determined at surgical re-exploration since the main non-invasive diagnostic tests have low accuracy. It would be desirable to have a high accuracy non-invasive diagnostic procedure. With this aim, we have assessed the utility of three-step immunoscintigraphy. Thirty patients were intravenously injected with biotinylated monoclonal antibodies MOv18 and B72.3, followed by avidin–streptavidin injection and finally by^111^In-biotin. Tumour recurrences were imaged 2 h post radioactivity injection. All patients underwent surgical re-exploration 3–4 days after immunoscintigraphy; the presence of tumour in the area of immunoscintigraphic uptake was evaluated in the biopsied material. Twenty-one patients studied were true-positive, five were true-negative, four were false-positive and none was false-negative. The diagnostic accuracy, positive predictive value and negative predictive value were 87%, 84% and 100% respectively. If these findings are confirmed in a larger number of patients, we expect immunoscintigraphy to be introduced as a cost-effective procedure in the follow-up of patients who have received surgery for ovarian carcinoma, since it promises to reliably identify patients who do not require surgical re-exploration, and guide biopsies when they are indicated. © 2000 Cancer Research Campaign


*British Journal of Cancer* (2000) **82**(3), 616–620
© 2000 Cancer Research Campaign
DOI: 10.1054/ bjoc.1999.0972, available online at http://www.idealibrary.com on IDEAL®

### Diagnosis of persistent ovarian carcinoma with three-step immunoscintigraphy

**P Magnani[1], F Fazio[1], C Grana[3], C Songini[1], L Frigerio[1], S Pecorelli[2], G Mangili[1], N Colombo[3], CDA Mariani[3] and G Paganelli[3]**

[1]INB-CNR, Institute H. S. Raffaele, University of Milan, Milan, Italy; [2]University of Brescia, Milan, Italy; [3]European Institute of Oncology, via Ripamonti 435, I-20132 Milan, Italy

**Summary** The diagnosis of recurrent ovarian carcinoma is usually determined at surgical re-exploration since the main non-invasive diagnostic tests have low accuracy. It would be desirable to have a high accuracy non-invasive diagnostic procedure. With this aim, we have assessed the utility of three-step immunoscintigraphy. Thirty patients were intravenously injected with biotinylated monoclonal antibodies MOv18 and B72.3, followed by avidin–streptavidin injection and finally by $^{111}$In-biotin. Tumour recurrences were imaged 2 h post radioactivity injection. All patients underwent surgical re-exploration 3–4 days after immunoscintigraphy; the presence of tumour in the area of immunoscintigraphic uptake was evaluated in the biopsied material. Twenty-one patients studied were true-positive, five were true-negative, four were false-positive and none was false-negative. The diagnostic accuracy, positive predictive value and negative predictive value were 87%, 84% and 100% respectively. If these findings are confirmed in a larger number of patients, we expect immunoscintigraphy to be introduced as a cost-effective procedure in the follow-up of patients who have received surgery for ovarian carcinoma, since it promises to reliably identify patients who do not require surgical re-exploration, and guide biopsies when they are indicated. © 2000 Cancer Research Campaign

**Keywords:** ovarian carcinoma; monoclonal antibodies; avidin–biotin

Ovarian cancer comprises approximately 4% of all new cases of cancer in women in North America and Western Europe. It is the most frequent cause of death among patients with gynaecological malignancies. Most patients are middle aged (40–70 years old) at the time of diagnosis (Ozols et al, 1992). Owing to the lack of clinical symptoms, primary ovarian carcinoma is often discovered in an advanced stage when the disease has spread beyond the ovaries throughout the peritoneal cavity.

At present, treatment of ovarian cancer consists of debulking surgery followed by platinum-based chemotherapy. Although the initial response to chemotherapy is high, the majority of patients with advanced-stage ovarian carcinoma develop residual or recurrent disease and approximately 70% of patients die within 5 years (Ozols et al, 1992).

The main diagnostic tests available to detect the presence of primary and residual or recurrent ovarian carcinoma are the serum marker assay CA125, physical examination, ultrasonography (US), computerized tomography (CT) and magnetic resonance imaging (MRI) (Morgan et al, 1992). However, these non-invasive techniques are unsatisfactory for a number of reasons. These methods cannot reliably detect small tumour deposits, diffuse peritoneal carcinosis or tumour in normal sized lymph nodes, all of which are primary modes of spread of ovarian cancer. Thus, the definitive diagnosis of primary ovarian carcinoma as well as the extent of recurrent or residual disease is usually determined at surgical re-exploration (second-look surgery) (Potter et al, 1980; Copeland et al, 1994). However, even a careful laparotomy may not detect all intra-abdominal disease present and cannot be used to evaluate extra-abdominal disease.

Furthermore, although about 50% of patients undergoing second-look are disease-free, about 60% of patients with a negative second look will have a recurrence in the peritoneal cavity (Cresman and Eddy, 1989; Podratz and Kinney, 1993). This suggests that non-detectable residual disease is present in the peritoneal cavity or retroperitoneal spaces at the time of second-look surgery (Gershenson et al, 1985), the sensitivity of which ranges from 76% to 88% (Rubin et al, 1991).

There is therefore a pressing need for a non-invasive test to detect the presence and determine the location and extent of occult disease. Such information would guide the surgeon during second-look laparotomy, whereas laparotomy could be avoided if the diagnostic test was negative.

After the development of monoclonal antibodies (mAbs) to tumour-associated antigens, immunoscintigraphy (ISG) has also been applied for the diagnosis and therapy of ovarian cancer (Rubin, 1993; Method et al, 1996). Moreover the recent development of multi-step procedures, in which the tumour is pretargeted, and the antibody and label are administered separately, has allowed background activity to be reduced, the tumour to normal tissue ratio to be increased, and ISG sensitivity to be improved (Crippa et al, 1991; Paganelli et al, 1991; Grana et al, 1996).

We have developed a three-step method, involving the avidin–biotin system, to target radioactive labels to mAbs already localized onto the tumour. Preclinical and clinical studies showed that multi-step avidin–biotin methods result in improved localization of radionuclides to tumours compared to the more

*Received 22 July 1998*
*Revised 5 November 1998*
*Accepted 20 November 1998*

*Correspondence to:* G Paganelli conventional 'one-step' approach with radioactive mAbs. The three steps we used are as follows: (i) tumour pretargeting by biotinylated anti-tumour mAbs; (ii) 'avidinization' of the biotinylated tumour-bound mAbs by administration of a molar excess of avidin; (iii) targeting the tumour by a fast clearing radioactive biotin derivative.

Numerous mAbs against ovarian cancer have been selected and characterized, such as the MOv18 mAb, which is expressed on the cell membrane of about 90% of human ovarian carcinomas (Grana et al, 1996) and exhibits a very restricted pattern of tissue distribution (Miotti et al, 1987; Mezzanzanica et al, 1988; Casalini et al, 1997), and the B72.3 mAb, which has been shown to have selective reactivity for a wide range of carcinomas, including ovarian cancer (Colcher et al, 1987). As the use of a cocktail of antibodies has been reported to increase the sensitivity of the method (Matzku et al, 1989), we used an association of MOv18 and B72.3 mAbs in this study.

The aim of this study was to evaluate the three-step pretargeting protocol in the diagnosis of residual or recurrent ovarian carcinoma. ISG was applied to evaluate the potential of this method to assess: (a) the absence/presence of residual tumour prior second-look laparotomy and (b) the potential of ISG in guiding the surgeon during second look.

### MATERIALS AND METHODS

### Patients

Thirty patients (38–78 years of age; mean 56.1), all with histologically confirmed ovarian cancer after primary surgery and chemotherapy treatment were enrolled in the study that was approved by the Ethical Committees of Scientific Institute HS Raffaele and European Institute of Oncology. Prior to study entry, all patients gave written informed consent.

Patients with a life expectancy less than 6 months or with renal or hepatic failure were excluded from the study. Twenty-eight patients were at stage III and two patients were at stage IV of the FIGO classification (20 G3 stage tumours and 11 G2 stage tumours). Histotype was serous in 17 patients, endometroid in seven, mucinous in two, clear cell in two and undifferentiated in two.

At the time of ISG, the serum marker CA125 was elevated in nine patients, normal in 17 patients and borderline in four.

Five patients had a positive CT/US scan, 12 patients were negative for the presence of neoplasia, whereas seven patients were doubtful on CT scan or US; in six patients imaging examinations were not performed.

All patients underwent second look within 1 week of ISG (mean 3 days). At surgery, samples from peritoneal tissues were taken. The mean number of biopsies/patient was 16 (range 5–35), taking care to sample areas which were positive at ISG.

### Reagents

Monoclonal antibody MOv18 (IgG1 subclass), which reacts with the most ovarian carcinomas, and B72.3 (IgG1 subclass), which recognizes a high molecular weight glycoprotein expressed by ovarian cancer cells, have been extensively described (Crippa et al, 1991; Larson et al, 1991). The biotinylation of mAbs has also been described (Magnani et al, 1996). Pure hen egg avidin and streptavidin were obtained from Società Prodotti Antibiotici (Milan, Italy). DTPA-conjugated biotin was purchased from Sigma (St Louis, MO, USA).

### Radiolabelling

DTPA-conjugated biotin was diluted in phosphate-buffered saline (PBS), pH 7.4, at a concentration of 2 mg ml$^{-1}$. The solution was sterilized by 0.22-μm Millipore filtration. $^{111}InCl_3$ was diluted in citrate buffer (0.02 M; pH 6.5) to 740 kBq ml$^{-1}$. The reagents were then mixed and allowed to react at room temperature for 10 min. More than 98% of $^{111}$In was bound to the conjugate, as shown by paper chromatography performed with Whatman no. 1 and bicarbonate buffer 0.05 M as liquid phase. The ability to bind avidin after labelling was verified by FPLC (Pharmacia, Sweden), by mixing $^{111}$In-biotin with an appropriate amount of avidin. No loss of reactivity was observed.

### Toxicity and immunogenicity

All patients were closely observed for 2 h following administration of avidin. Blood samples (10 ml) were obtained in appropriate tubes just before the administration of avidin, and 20–25 days after the injection. All samples were sent for routine blood tests and to assess the human anti-mouse immunoglobulin (HAMA) and anti-avidin response (HAAR).

The induction of NAMA antibodies was investigated using an enzyme-linked immunosorbent assay (ELISA) system (Seccamani et al, 1989) in 15 patients. In these patients, avidin immunogenicity was studied on microwell plates coated with avidin or streptavidin separately. The plates were saturated for 1 h with PBS-3% bovine serum albumin (BSA). Diluted human serum was added and incubated for 1 h at 37°C. After five washes, the binding of human anti-avidin antibodies was revealed with horseradish peroxidase-conjugated rabbit anti-human Ig antibodies (Dako) diluted 1:1000 for 45 min at 37°C. After six washes, the enzymatic reaction was developed with a chromogenic substrate (*O*-phenylenediamine; Sorin Biomedica, Saluggia, Italy) for 10 min and blocked by addition of 1 M $H_2SO_4$. The optical density reading was 492 nm.

### ISG study

The three-step protocol used has been previously described (Grana et al, 1996). Briefly, in 15 patients, 1 mg of biotinylated mAb MOv18 was injected intravenously (i.v.) over 2 min (first step). After 24–36 h, 1 mg of unlabelled avidin was injected i.v. over 2 min followed by an additional 5 mg of streptavidin 15 min later (second step). The aims of these two avidin administrations were, respectively, to precipitate circulating biotinylated antibodies (1 mg) and subsequently to target the biotinylated MAbs bound on tumour cells (5 mg).

In 15 patients, 1 mg of biotinylated mAb MOv18 plus 1 mg of biotinylated B72.3 were injected i.v. over 2 min (first step). After 24–36 h, 2 mg of unlabelled avidin was injected i.v. over 2 min, followed by additional 5 mg of streptavidin 15 min later (second step).

In all patients, 200 μg of $^{111}$In-biotin (111–185 MBq) was injected i.v. 24 h after administration of the cold avidin in 3 ml of saline solution in a bolus injection (third step).

© 2000 Cancer Research Campaign

After [111]In-biotin injection, single photon emission tomography (SPECT) studies (64 × 64 pixel matrix, 64 projections over 360°) of the thorax, abdomen and whole body were performed. Imaging was performed shortly (1–3 h) after injection of the radiolabel, as background radioactivity levels were low, due to the three-step technique employed and fast elimination of the small labelled biotin molecule.

Images were obtained using rotating gamma-cameras (7500 Orbiter Siemens and Starcam 400 GE), equipped with medium energy collimators and by selecting two 15% energy windows centred over the 173 and 247 keV photopeaks of [111]In.

Tomographic images were reconstructed using a filtered back projection algorithm and Hann filter (cut-off 0.5 pixel$^{-1}$). Planar and tomographic images were evaluated for the presence or absence of pathological tracer accumulation by two independent observers who were unaware of the clinical problem and biochemical tumour marker levels. In case of disagreement, the images were reviewed by a third reader. ISG results were classified as true-positive (TP), true-negative (TN), false-positive (FP) and false-negative (FN) according to the histological diagnosis.

### RESULTS

No toxicity was observed after reagent administration. Of the 15 patients tested, two (13%) developed an antibody response to mouse immunoglobulins, whereas four (27%) patients demonstrated anti-avidin and five (33%) patients anti-streptavidin antibodies. There was 100% agreement in the evaluation of ISG results between the two observers. The results are summarized in Table 1.

There were no differences in results between the two groups of patients receiving only one kind of antibody or the cocktail of two antibodies: in fact neither in the group who received one antibody nor in the other patients did we observe any FN results. Moreover, in the group of patients with the cocktail of mAbs we observed a smaller number of FPs than in the other group.

Pathological tracer accumulation was observed by ISG in 25 (83%) of 30 patients; in 21 patients second-look surgery confirmed the presence of recurrent tumour: 19 had microscopic disease whereas two had gross macroscopic tumour. In the nine patients with negative histopathological findings, ISG was correctly negative in five patients. According to these data, the results were as follows: 21 TP, five TN and four FP; none was FN. See Figures 1 and 2 as examples of three-step ISG.

CA125 serum level indicated the presence of a recurrent ovarian carcinoma in nine (29%) patients, all recognized as TP by ISG. In four (13%) patients a borderline CA125 serum level was associated with one TN and three TP results, whereas the 17 (57%) normal CA125 serum levels consisted of four TN, nine TP and four FP.

**Table 1** Patient data and results

| Patient no. | Age | Stage | CA125 | US/CT | ISG | mAb |
|---|---|---|---|---|---|---|
| 1 | 58 | IIIC | 3.0 | N | TN | Mov18 |
| 2 | 48 | IIIB | 2.5 | N | TN | Mov18 |
| 3 | 60 | IIIC | 18.0 | NP | TP | Mov18 |
| 4 | 60 | IIIC | 33.0 | N | TP | Mov18 |
| 5 | 38 | IV | 35.0 | N | TP | Mov18 |
| 6 | 61 | IIIC | 1.9 | NP | TN | Mov18 |
| 7 | 62 | IIIC | 27.0 | NP | TP | Mov18 |
| 8 | 65 | IIIC | 25.0 | NP | FP | Mov18 |
| 9 | 55 | IIIC | 6.6 | N | FP | Mov18 |
| 10 | 53 | IIIB | 34.0 | NP | TN | Mov18 |
| 11 | 78 | IIIC | 35.0 | D | TP | Mov18 |
| 12 | 51 | IIIC | 27.0 | N | TP | Mov18 |
| 13 | 43 | IIIC | 25.0 | NP | TP | Mov18 |
| 14 | 64 | IIIC | 22.0 | N | TP | Mov18 |
| 15 | 61 | IV | 10.0 | N | FP | Mov18 |
| 16 | 44 | IIIC | 11.0 | P | TP | Mov18 + B72.3 |
| 17 | 59 | IIIA | 620.0 | D | TP | Mov18 + B72.3 |
| 18 | 47 | IIIC | 4.9 | P | TP | Mov18 + B72.3 |
| 19 | 70 | IIIC | 169.0 | D | TP | Mov18 + B72.3 |
| 20 | 48 | IIIB | 530.0 | P | TP | Mov18 + B72.3 |
| 21 | 56 | IIIC | 300.0 | P | TP | Mov18 + B72.3 |
| 22 | 57 | IIIC | 4.0 | N | FP | Mov18 + B72.3 |
| 23 | 50 | IIIC | 7.8 | D | TP | Mov18 + B72.3 |
| 24 | 43 | IIIB | 18.0 | D | TP | Mov18 + B72.3 |
| 25 | 66 | IIIC | 6.9 | N | TN | Mov18 + B72.3 |
| 26 | 53 | IIIC | 239.0 | N | TP | Mov18 + B72.3 |
| 27 | 53 | IIIC | 93.0 | D | TP | Mov18 + B72.3 |
| 28 | 66 | IIIC | 52.0 | D | TP | Mov18 + B72.3 |
| 29 | 54 | IIIC | 73.0 | P | TP | Mov18 + B72.3 |
| 30 | 53 | IIIC | 1200.0 | N | TP | Mov18 + B72.3 |

FP: false-positive; TN: true-negative; TP: true-positive; N: negative; P: positive; D: doubtful; NP: not performed. CA125 range: 0–35 μ ml$^{-1}$.

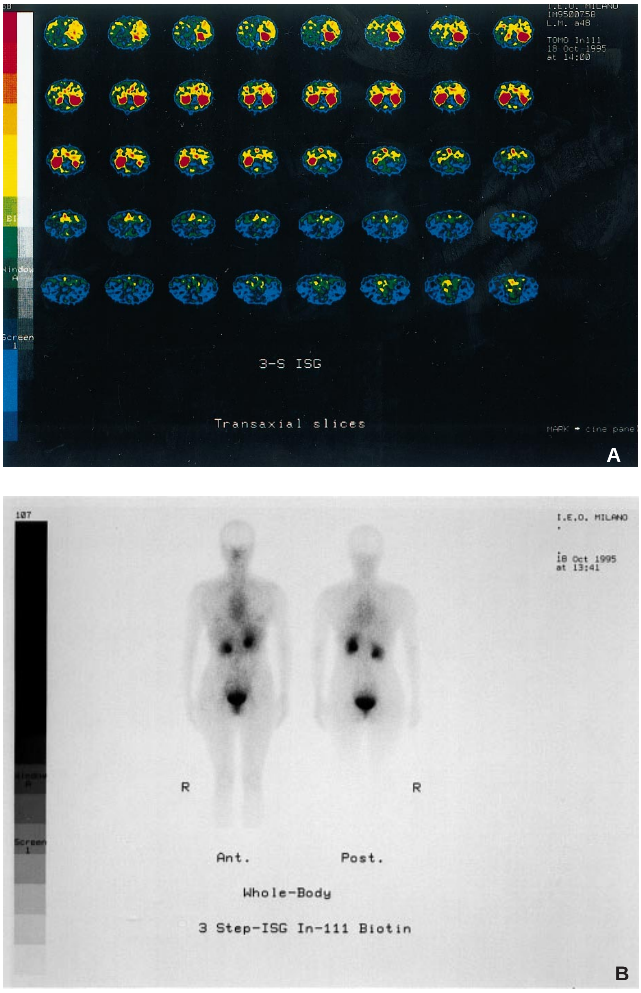

**Figure 1** (**A**) ISG study (transaxial slices) of patient no. 18 (LM, 48 years old, IEO); (**B**) pathological tracer uptake is clearly evident into the whole abdomen, particularly in mesogastrium, representative of peritoneal carcinosis. These data were then confirmed during the second-look

*© 2000 Cancer Research Campaign*

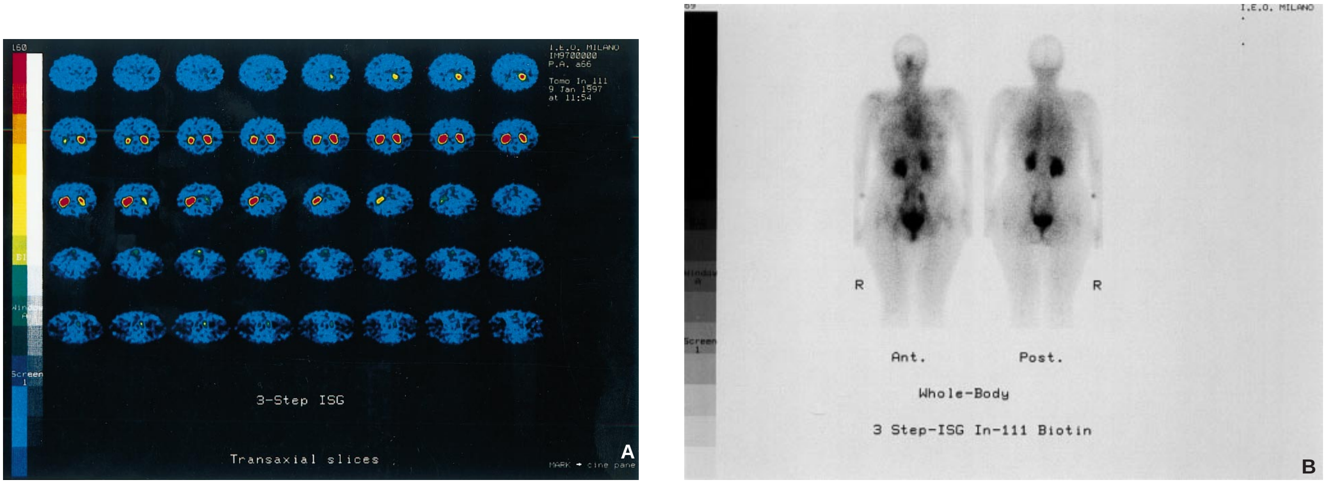

**Figure 2** ISG performed in patient no. 25 (PA, 66 years old, IEO). This patient was treated with surgery for stage IIIC ovarian carcinoma, followed by chemotherapy, then underwent ISG; in these images (**A**: transaxial slices) there is no evidence of pathological uptake of the tracer (**B**). This negative result was then confirmed at surgery

**Table 2** CA125 serum level and ISG comparison

| | ISG P | ISG N |
|---|---|---|
| CA125 P | 9 (TP) | 0 |
| CA125 N | 9 (TP); 4 (FP) | 5 (TN) |
| CA125 D | 3 (TP) | 1 (TN) |

FP: false-positive; TN: true-negative; TP: true-positive. CA125 range: 0–35 $\mu$ ml$^{-1}$.

CT/US scan indicated the presence of recurrent disease in five (16%) patients, all of whom were TP. All the seven (23%) doubtful CT cases turned out to be TP by ISG. Among 12 (39%) patients in whom CT/US examination did not reveal the presence of recurrent disease, three were TN, six were TP and three were FP.

Combining the CA125 serum level and the CT/US information, a correct diagnosis was obtained in 17 (57%) patients, whereas 13 (42%) patients were erroneously evaluated. These 13 patients consisted of 12 TP and one TN by ISG.

Combining the CA125 serum level and the ISG information, a correct diagnosis was obtained in 26 (84%) patients (21 TP and five TN), whereas four (13%) patients were FP (Table 2).

The diagnostic accuracy, positive predictive value and negative predictive value of ISG were 87%, 84% and 100% respectively.

### DISCUSSION

Due to lack of clinical symptoms, primary ovarian carcinoma is often discovered at an advanced stage when disease has spread beyond the ovaries to the peritoneal cavity and other organs. For this reason first laparotomy is not only important therapeutically but also prognostically. In all the more advanced stages of ovarian carcinoma, chemotherapy is a standard adjuvant to surgical treatment.

Combined physical examination, serum CA125 assay and CT scan of the abdomen and pelvis is used to assess disease status in patients who respond to primary cytoreductive surgery and adjuvant chemotherapy. Unfortunately, the accuracy of these non-surgical techniques in detecting persistent or recurrent disease is poor; and the extent of recurrent or residual disease is generally determined by surgical re-exploration. Such second-look exploration is useful for determining the efficacy of treatment, identifying additional sites of recurrence and planning additional therapy and tumour debulking.

Immunoscintigraphy promises to provide more reliable non-invasive detection and localization of persistent or recurrent tumour. Recent studies (Lown et al, 1995) reported that the technique has sensitivity of 72% and 71% in detecting suspected recurrence and carcinomatosis respectively. However, after injection of directly labelled mAb, image interpretation is often difficult because of non-specific bone marrow, vascular and liver activity uptake (Crippa et al, 1991). For example, a limitation of $^{111}$In-antibody scanning is its insensitivity to subphrenic, perihepatic and perisplenic peritoneal tumour because physiological uptake of $^{111}$In-mAb by the liver and spleen obscures findings from adjacent peritoneal tumour. In carcinoembryonic antigen (CEA)-positive tumours, gliomas, lung cancers and neuroendocrine tumours, a new ISG method involving the avidin–biotin pretargeting approach (Colombo et al, 1993; Dosio et al, 1994; Paganelli et al, 1994) has provided good results, attributable in part to the absence of physiological hepatic and splenic uptake, as well as with the use of bispecific mAb, as Peltier stated in his work (Peltier et al, 1993).

Important advantages of the pretargeting/ISG approach are that circulating antibodies are quickly removed by avidin in the second step, while the radiolabelled biotin, that is administered subsequently, either binds to the tumour-bound antibody or is eliminated quickly. This allows images with low background activity to be obtained. Use of the SPECT technique further improves ability to detect small quantities of residual disease.

We employed this pretargeting approach in 30 ovarian carcinoma patients enrolled for second-look exploration. We used mAb MOv18, which is expressed on the cell membrane of about 90% of ovarian carcinomas. In addition, since the second and third step of the three-step protocol can be common to all protocols, other potentially useful mAbs can be injected in sequence or in combination as the first step, and in fact we injected the cocktail of MOv18 and B72.3 mAbs in 15 of these patients.

We did not find any significant differences in the two groups of

© 2000 Cancer Research Campaign patients; however, we think that the administration of a cocktail of antibodies could result in a targeting of a higher number of tumour cells and in an increased sensitivity of the method, despite loss of specificity. This could increase the number of FP but avoid the FN.

Comparison of our ISG results with the second-look biopsy findings showed that ISG gave 21 TP, five TN and four FP, so that overall diagnostic accuracy was 87%. The relatively high number of FP (13%) is probably attributable to the presence of inflammatory tissue, where there is an aspecific uptake due to the inflammatory process itself. All the 13 patients with FN result by combined CA125 and CT/US were correctly diagnosed by ISG. It is also noteworthy that for the series as a whole, the ISG technique had a particularly high negative predictive value.

We conclude, therefore, that ISG can improve the sensitivity of second-look surgery (particularly laparoscopy), as the surgeon can direct his attention to, and sample sites of, tracer accumulation revealed by the ISG procedure. Note that although laparotomy is currently considered the best method for evaluating ovarian recurrences, its sensitivity is in the range 76–88% (Paganelli et al, 1991). Our results also suggest that a negative ISG result, associated with low CA125 serum levels and negative CT/US findings, can be interpreted as real absence of recurrent disease, thus reducing the need for second-look laparotomy.

If these findings are confirmed in a larger number of patients, we expect ISG to be introduced as a cost-effective procedure in the follow-up of patients who have received surgery for ovarian carcinoma, since it promises to reliably identify patients who do not require second-look laparotomies, and guide biopsies when they are indicated. We believe our results justify a randomized clinical trial to compare survival in patients who undergo second-look laparotomy alone with survival in patients who undergo ISG guided second-look laparotomy.

### ACKNOWLEDGEMENTS

This work was supported in part by grant from the Associazione Italiana per la Ricerca sul Cancro (AIRC).

### REFERENCES

Casalini P, Luison E, Menard S, Colnaghi MI, Paganelli G and Canevari S (1997) Tumor pretargeting: role of avidin/streptavidin on monoclonal antibody internalization. *J Nucl Med* **38:** 1378–1381

Colcher D, Esteban J and Carasquillo JA (1987) Complementation of intracavitary and intravenous administration of a monoclonal antibody (B72.3) in patients with carcinoma. *Cancer Res* **47:** 4218–4224

Colombo P, Paganelli G, Magnani P, Songini C, Fazio F and Faglia G (1993) Immunoscintigraphy with anti-chromogranin A antibodies in patients with endocrine/neuroendocrine tumors. *J Endocrinol Invest* **16:** 841–843

Copeland LJ, Vaccarello L and Lewandowski GS (1994) Second-look laparotomy in epithelial ovarian cancer. *Obst Gynecol Clin North Am* **21:** 155–166

Cresman WT and Eddy GL (1989). Prognostic factors in relation to second look laparotomy in ovarian cancer. *Ballières Clin Obstet Gynecol* **3:** 183–190

Crippa F, Buraggi GL, DiRe E, Gasparrini M, Seregni E, Canevari S, Gadina M, Presti M, Marini A and Seccamani E (1991). Radioimmunoscintigraphy of ovarian cancer with the MOv 18 monoclonal antibody. *Eur J Cancer* **27:** 724–772

Dosio F, Magnani P, Paganelli G, Samuel A, Chiesa G and Fazio F (1994) Three-step tumor pre-targeting in lung cancer. *J Nucl Biol Med* **37:** 228–233

Gershenson DM, Copeland LJ, Wharton JT, Atkinson EN, Sneige N, Edwards CL and Rutledge FN (1985) Prognosis of surgically determined complete responders in advanced ovarian cancer. *Cancer* **55:** 1129–1135

Grana C, Chinol M, Magnani P, Corti A, Sidoli A, Siccardi AG and Paganelli G (1996) In vivo tumor targeting based on the avidin–biotin system. *Tumor Targeting* **2:** 230–239

Larson SM, Carrasquillo JA, Colcher DC, Yokoyama K, Reynolds JC, Bacharach SA et al (1991) Estimates of radiation absorbed dose for intraperitoneally administered Iodine-131 radiolabeled B72.3 monoclonal antibody in patients with peritoneal carcinomatoses. *J Nucl Med* **32**: 1661–1667

Lown RN, Carter WD, Saleh F and Sigeti JS (1995) Ovarian cancer: comparison of findings with perfluorocarbon-enhanced MR imaging, In-111-CYT-103 immunoscintigraphy and CT. *Radiology* **195**: 391–400

Magnani P, Paganelli G, Songini C, Samuel A, Sudati F, Siccardi AG and Fazio F (1996) Pretargeted immunoscintigraphy in patients with medullary thyroid carcinoma. *Br J Cancer* **74**: 825–831

Matzku U, Kirchgessner H, Schmid U, Temponi M and Ferrone S (1989) Melanoma targeting with a cocktail of monoclonal antibodies to distinct determinants of the human HMW-MAA. *J Nucl Med* **30**: 390–397

Method MW, Serafini AN, Averette HE, Rodriguez M, Penalver MA and Sevin B (1996) The role of radioimmunoscintigraphy and computed tomography scan prior to reassessment laparotomy of patients with ovarian carcinoma. *Cancer* **77**: 2286–2293

Mezzanzanica D, Canevari S and Menard S (1988) Human ovarian carcinoma lysis by cytotoxic T-cells targeted by bispecific monoclonal antibodies: analysis of the antibody components. *Int J Cancer* **41**: 609–615

Miotti S, Canevari S and Menard S (1987) Characterization of human ovarian carcinoma associated antigens defined by novel monoclonal antibodies with tumor restricted specificity. *Int J Cancer* **39**: 297–303

Morgan MA, Noumoff JS, King S and Mikuta JJ (1992) A formula for predicting the risk of a positive second-look laparotomy in epithelial ovarian cancer: implications for a randomized trial. *Obstet Gynecol* **80**: 944–948

Ozols RF, Rubin SC, Dembo A and Robboy SJ (1992) Epithelial ovarian cancer. In: *Principles and Practice of Gynecologic Oncology*, Hoskins WJ, Young R and Perez C (ed), pp. 731–781. Lippincott: Philadelphia.

Paganelli G, Magnani P, Zito F, Villa E, Sudati F, Lopalco L, Rossetti C, Malcovati M, Chiolerio F, Seccamani E, Siccardi AG and Fazio F (1991) Three-step monoclonal antibody tumor targeting in carcinoembryonic antigen-positive patients. *Cancer Res* **51**: 5960–5966

Paganelli G, Magnani P, Zito F, Lucignani G, Sudati F, Truci G, Motti E, Terreni M, Pollo B, Giovanelli M, Canal N, Scotti G, Comi G, Koch P, Maecke HR and Fazio F (1994) Pre-targeted immunodetection in glioma patients: tumour localization and single-photon emission tomography imaging of [99mTc]PnAO-biotin. *Eur J Nucl Med* **21**: 314–321

Peltier P, Curtet C, Chatal JF, Le Doussal JM, Daniel G, Aillet G, Gruaz-Guyon A, Barbet J and Delaage M (1993) Radioimmunodetection of medullary thyroid cancer using a bispecific anti-CEA/anti-Indium-DTPA and an Indium-111-labelled DTPA dimer. *J Nucl Med* **34**: 1267–1273

Podratz KC and Kinney WK (1993) Second-look operation in ovarian cancer. *Cancer* **71**: 1551–1558

Potter ME, Hatch KD and Soong SJ (1980) Second look laparotomy and salvage therapy: research modality only? *Gynecol Oncol* **44**: 3

Rubin SC, Hoskins WJ, Saigo PE, Chapman D, Hakes TB and Markman M (1991) Prognostic factors for recurrence following negative second-look laparotomy in ovarian cancer patients treated with platinum based chemotherapy. *Gynecol Oncol* **42**: 137–141

Rubin SC (1993) Monoclonal antibodies in the management of ovarian carcinoma: a clinical perspective. *Cancer* **71**: 1602–1612

Seccamani E, Tattanelli M, Mariani M, Spranzi E, Scassellati GA and Siccardi AG (1989) A simple qualitative determination of human antibodies to murine immunoglobulins (HAMA) in serum sample. *Nucl Med Biol* **2**: 167–170

© 2000 Cancer Research Campaign

## References

[PDF_00431] Casalini P., Luison E., Ménard S., Colnaghi M. I., Paganelli G., Canevari S. (1997). Tumor pretargeting: role of avidin/streptavidin on monoclonal antibody internalization.. J Nucl Med.

[PDF_00434] Colcher D., Esteban J., Carrasquillo J. A., Sugarbaker P., Reynolds J. C., Bryant G., Larson S. M., Schlom J. (1987). Complementation of intracavitary and intravenous administration of a monoclonal antibody (B72.3) in patients with carcinoma.. Cancer Res.

[PDF_00437] Colombo P., Paganelli G., Magnani P., Songini C., Fazio F., Faglia G. (1993). Immunoscintigraphy with anti-chromogranin A antibodies in patients with endocrine/neuroendocrine tumors.. J Endocrinol Invest.

[PDF_00440] Copeland L. J., Vaccarello L., Lewandowski G. S. (1994). Second-look laparotomy in epithelial ovarian cancer.. Obstet Gynecol Clin North Am.

[PDF_00442] Creasman W. T., Eddy G. L. (1989). Prognostic factors in relation to second-look laparotomy in ovarian cancer.. Baillieres Clin Obstet Gynaecol.

[PDF_00444] Crippa F., Buraggi G. L., Di Re E., Gasparini M., Seregni E., Canevari S., Gadina M., Presti M., Marini A., Seccamani E. (1991). Radioimmunoscintigraphy of ovarian cancer with the MOv18 monoclonal antibody.. Eur J Cancer.

[PDF_00448] Dosio F., Magnani P., Paganelli G., Samuel A., Chiesa G., Fazio F. (1993). Three-step tumor pre-targeting in lung cancer immunoscintigraphy.. J Nucl Biol Med.

[PDF_00450] Gershenson D. M., Copeland L. J., Wharton J. T., Atkinson E. N., Sneige N., Edwards C. L., Rutledge F. N. (1985). Prognosis of surgically determined complete responders in advanced ovarian cancer.. Cancer.

[PDF_00456] Larson S. M., Carrasquillo J. A., Colcher D. C., Yokoyama K., Reynolds J. C., Bacharach S. A., Raubitchek A., Pace L., Finn R. D., Rotman M. (1991). Estimates of radiation absorbed dose for intraperitoneally administered iodine-131 radiolabeled B72.3 monoclonal antibody in patients with peritoneal carcinomatoses.. J Nucl Med.

[PDF_00460] Low R. N., Carter W. D., Saleh F., Sigeti J. S. (1995). Ovarian cancer: comparison of findings with perfluorocarbon-enhanced MR imaging, In-111-CYT-103 immunoscintigraphy, and CT.. Radiology.

[PDF_00463] Magnani P., Paganelli G., Songini C., Samuel A., Sudati F., Siccardi A. G., Fazio F. (1996). Pretargeted immunoscintigraphy in patients with medullary thyroid carcinoma.. Br J Cancer.

[PDF_00466] Matzku S., Kirchgessner H., Schmid U., Temponi M., Ferrone S. (1989). Melanoma targeting with a cocktail of monoclonal antibodies to distinct determinants of the human HMW-MAA.. J Nucl Med.

[PDF_00469] Method M. W., Serafini A. N., Averette H. E., Rodriguez M., Penalver M. A., Sevin B. U. (1996). The role of radioimmunoscintigraphy and computed tomography scan prior to reassessment laparotomy of patients with ovarian carcinoma. A preliminary report.. Cancer.

[PDF_00473] Mezzanzanica D., Canevari S., Ménard S., Pupa S. M., Tagliabue E., Lanzavecchia A., Colnaghi M. I. (1988). Human ovarian carcinoma lysis by cytotoxic T cells targeted by bispecific monoclonal antibodies: analysis of the antibody components.. Int J Cancer.

[PDF_00476] Miotti S., Canevari S., Ménard S., Mezzanzanica D., Porro G., Pupa S. M., Regazzoni M., Tagliabue E., Colnaghi M. I. (1987). Characterization of human ovarian carcinoma-associated antigens defined by novel monoclonal antibodies with tumor-restricted specificity.. Int J Cancer.

[PDF_00479] Morgan M. A., Noumoff J. S., King S., Mikuta J. J. (1992). A formula for predicting the risk of a positive second-look laparotomy in epithelial ovarian cancer: implications for a randomized trial.. Obstet Gynecol.

[PDF_00489] Paganelli G., Magnani P., Zito F., Lucignani G., Sudati F., Truci G., Motti E., Terreni M., Pollo B., Giovanelli M. (1994). Pre-targeted immunodetection in glioma patients: tumour localization and single-photon emission tomography imaging of [99mTc]PnAO-biotin.. Eur J Nucl Med.

[PDF_00485] Paganelli G., Magnani P., Zito F., Villa E., Sudati F., Lopalco L., Rossetti C., Malcovati M., Chiolerio F., Seccamani E. (1991). Three-step monoclonal antibody tumor targeting in carcinoembryonic antigen-positive patients.. Cancer Res.

[PDF_00494] Peltier P., Curtet C., Chatal J. F., Le Doussal J. M., Daniel G., Aillet G., Gruaz-Guyon A., Barbet J., Delaage M. (1993). Radioimmunodetection of medullary thyroid cancer using a bispecific anti-CEA/anti-indium-DTPA antibody and an indium-111-labeled DTPA dimer.. J Nucl Med.

[PDF_00498] Podratz K. C., Kinney W. K. (1993). Second-look operation in ovarian cancer.. Cancer.

[PDF_00500] Potter M. E., Hatch K. D., Soong S. J., Partridge E. E., Austin J. M., Shingleton H. M. (1992). Second-look laparotomy and salvage therapy: a research modality only?. Gynecol Oncol.

[PDF_00502] Rubin S. C., Hoskins W. J., Saigo P. E., Chapman D., Hakes T. B., Markman M., Reichman B., Almadrones L., Lewis J. L. (1991). Prognostic factors for recurrence following negative second-look laparotomy in ovarian cancer patients treated with platinum-based chemotherapy.. Gynecol Oncol.

[PDF_00506] Rubin S. C. (1993). Monoclonal antibodies in the management of ovarian cancer. A clinical perspective.. Cancer.

[PDF_00508] Seccamani E., Tattanelli M., Mariani M., Spranzi E., Scassellati G. A., Siccardi A. G. (1989). A simple qualitative determination of human antibodies to murine immunoglobulins (HAMA) in serum samples.. Int J Rad Appl Instrum B.

